# Social cognition in Wilson’s disease: A new phenotype?

**DOI:** 10.1371/journal.pone.0173467

**Published:** 2017-04-06

**Authors:** Elodie Peyroux, Nelly Santaella, Emmanuel Broussolle, Caroline Rigard, Emilie Favre, Anne-Sophie Brunet, Muriel Bost, Alain Lachaux, Caroline Demily

**Affiliations:** 1 Genopsy–Center for the Diagnosis and Management of Genetic Psychiatric Disorders, Le Vinatier Hospital, Lyon, France; 2 University Department of Rehabilitation (SUR/CL3R), Le Vinatier Hospital, Lyon, France; 3 Lumière–Lyon 2 University, Lyon, France; 4 Department of Neurology C, Pierre Wertheimer Neurological Hospital, *Hospices Civils de Lyon*, Lyon, France; 5 Center of Cognitive Neurosciences, UMR 5229, CNRS, Lyon, France; 6 Claude Bernard–Lyon 1 University, Lyon, France; 7 French National Center for Wilson’s Disease, *Hospices Civils de Lyon*, Lyon, France; 8 Department of Pediatric Gastroenterology, Hepatology and Nutrition, Children’s Hospital of Lyon, *Hospices Civils de Lyon*, Lyon, France; 9 Laboratory of Inherited Metabolic Diseases, CBPE, Lyon, France; 10 Laboratory of Trace Element and Toxic Metal Analysis, Edouard Herriot Hospital, Lyon, France; Universitatsklinikum Tubingen, GERMANY

## Abstract

Studies focusing on neuropsychological impairments in Wilson’s disease (WD) have highlighted that patients showing neurological signs present significant deficits in a wide range of cognitive domains. Attentional and executive impairments have also been described in people with hepatic WD. However, social cognition abilities, *i*.*e*. cognitive processes required to perceive the emotions, intentions and dispositions of other people, have not been clearly investigated in WD. In this study we examined the social cognitive functioning in 19 patients with WD depending on their clinical status–Neurological versus Non-Neurological (“hepatic”) forms–compared to 20 healthy controls. For the very first time, results highlighted that patients with WD had significant impairments in the three major components of social cognition: emotion recognition, Theory of Mind and attributional style. However, these deficits differ depending on the form of the disease: patients with neurological signs showed a wide range of deficits in the three components that were assessed–results notably revealed impairments in recognizing “fear”, “anger”, and “disgust”, a significant Theory of Mind deficit and an “aggression bias”–whereas Non-Neurological patients only showed deficits on test assessing attributional bias, with a trend to react more “aggressively” to ambiguous social situations than healthy controls, as observed in Neurological WD patients, and a specific impairment in “anger” recognition. Our findings are discussed in the light of both neurocognitive impairments and brain damages, and especially those affecting the basal ganglia, as observed in people with WD.

## Introduction

Wilson’s disease (WD) is a rare autosomal recessive inherited disease, involving mutation of the ATP7B gene on chromosome 13, which induces an adenosine triphosphate (ATP) production deficiency [1,2]. WD is characterized by copper accumulation in various organs including the liver, brain, cornea and kidney. Consequently, clinical presentations may be highly variable with either predominant hepatic, neurological or psychiatric symptoms. Cognitive deterioration in WD has been described since the first cases reported by Wilson in 1912, but only few studies have examined neuropsychological profiles in WD patients. Even if the criteria for the characterization of the WD patient clinical status–with or without neurological signs–differ from one study to the other, they seem to clearly help distinguish WD patients who show neurological signs–hereafter referred to as “Neurological WD”–and those who do not–“Non-Neurological WD”. Using MRI criteria, Seniów *et al*. showed that Non-Neurological WD patients did not present with any cognitive impairment compared to healthy controls [3]. Other studies using clinical criteria–the absence of motor signs–disclosed attentional and executive impairments in Non-Neurological WD patients [4,5]. Neurological WD is indeed associated with significant deficits in a wide range of cognitive functions including basic processes such as motor functioning, some aspects of memory, visuospatial processing, and above all executive functions [3,6–8]. It is also well-established that patients suffering from Neurological WD may show impulsivity, irritability and behavioral abnormalities [9] or other psychiatric manifestations [10].

In contrast to the above-mentioned cognitive functions, some aspects of social cognition, defined as “the mental operations underlying social interactions” [11] or as “the ability to construct representations of the relation between oneself and others and to use those representations flexibly to guide social behavior” [12], have not, to our knowledge, been clearly described in WD.

Social cognitive processes belong to the field of social neurosciences, and are the interface between basic perception processes and behavior in the social world [13]. In psychiatry, social cognition has been studied since the 1990s in populations with social dysfunction as a central characteristic and diagnostic criterion, such as people with schizophrenia or autism disorders. In neurological disorders, assessment of social cognition during the initial standard neurological examination is less usual. However, depending on the neurological disorders, social cognitive impairment may be either a prominent clinical symptom, a core feature of the early stages of some dementia, or a very subtle, hard-to-detect deficit but be useful to understand the patients’ functional disabilities [14]. Moreover, the importance of a clinical assessment of social cognitive functions in most of the neurological, developmental, psychiatric, and genetic disorders is now formally recognized in the fifth edition of the Diagnostic and Statistical Manual for mental disorders (DSM-5) [15].

In WD, personality, emotional, and behavioral disorders are frequently encountered [16]. In the literature, only one study investigated emotion recognition in patients with WD but without differentiating Neurological from Non-Neurological WD cases. Results showed that patients performed poorly compared to normal controls [17]. However, social cognition is not limited to emotion recognition and is made of several components such as: emotional processing, *i*.*e*. the ability to identify and recognize emotions through facial expressions, gestures and voice tone; Theory of Mind (ToM), which is the ability to attribute mental states–beliefs, intents, desires, pretending, knowledge, etc.–to oneself and others and to understand that others have beliefs, desires, and intentions that differ from one’s own; social perception and knowledge, which can be defined as decoding and interpreting social cues from others, taking the social context into account, and being aware of social rules, roles, and goals; and attributional style, which refers to how people explain the causes of positive and negative events–by attributing the event to oneself, somebody else or the context of the situation. These dysfunctions lead to dysfunctions of social behavior, and are based on neuroanatomical disturbances which should not be understood in terms of specific structures, but rather in terms of their interaction in large-scale networks [18].

The main goal of the present study is to characterize, for the very first time, social cognitive functioning in WD patients compared to healthy controls, by focusing on three major components of social cognition, namely facial emotion recognition, ToM, and attribution bias. We made the hypothesis that if social cognition processes are impaired in WD, it will be particularly obvious in Neurological WD.

## Materials and method

This project was approved by the ethical committee of the Hospices Civils de Lyon. The study was carried out in accordance with the Declaration of Helsinki, and all participants provided written informed consent to participate in the study.

### Participants

Nineteen patients with WD were recruited at the Hospices Civils de Lyon (CMR Wilson) in Lyon, France, and were compared to twenty healthy participants matched one by one based on age, gender, and level of education. Recruitment procedure took place between November 2013 and April 2015. All subjects were French native speakers and presented with normal or corrected-to-normal visual acuity.

Diagnosis of WD in all patients was established by copper metabolism and ceruloplasmin data and by ATP7B gene mutation analyses. At the time of the investigation, the patients’ neurological and hepatic conditions were considered stable. Exclusion criteria for the present study were substance abuse, depressive comorbidities (Beck Depression Inventory [19]), or history of head injury or cerebrovascular insult. WD patients were subdivided into two subgroups according to the presence (Neurological WD) or absence (Non-Neurological WD) of clinical neurological symptoms. Patient evaluation was based on careful and extensive clinical examination by a movement disorder specialist and WD expert (EB). Patients were classified as Non-Neurological WD when neurological examination did not reveal any sign of dystonia, akinesia, rigidity, gait disturbance, cerebellar signs, dysarthria, occulomotor signs or any kind of tremor at the time of the study, nor in any assessment from the diagnosis to the beginning of the study.

Almost all Non-Neurological WD patients underwent ophthalmological examination (slit lamp) to determine the presence of a Kayser-Flescher ring (KF). Only two of them presented a KF at the time of the diagnosis. These patients were included in the present study respectively 5 and 10 years after the initial ophthalmological examination. Both presented with liver failure and were thus treated with liver transplant within a month, after diagnosis. None presented with neurological signs at diagnosis or follow-up examinations. In addition, both had normal brain MRI. In the Neurological WD group, eight out of ten patients were examined with a slit lamp and six showed Kayser-Fleischer rings. Eight out of nine patients in the Non-Neurological group were investigated with brain MRI; all results were normal. In the Neurological WD group, all patients were investigated and presented with the classical MRI WD profile with abnormal signal in the basal ganglia and brain stem.

At the time of the investigation, all WD patients were on long-term drug therapy (with copper chelators or zinc). Three patients in each group were treated with liver transplant. The three patients from the Non-Neurological group were transplanted for liver failure. Two patients in the Neurological group were transplanted for liver failure and one for neurological disability. All transplanted patients were receiving standard immunosuppressant drug therapy after liver transplant.

Healthy participants were recruited from the community through advertising. They had no history of mental health problems, head injuries, neurological disorders or family history of WD. The two patient groups and the control group were matched on age, gender and educational level. The precise characteristics of patients and controls are shown in Table 1.

**Table 1:** Characteristics of Neurological WD patients, Non-Neurological WD patients and healthy controls.

					Neurologic WD	Non Neurologic WD	Controls	*p*-value
							
					N = 10	N = 9	N = 20	
					Mean (SD)	Mean (SD)	Mean (SD)	
Age at onset (years)					19.3 (3.5)	10.44 (4.53)	-	*p* = .0002
Age at time of the study (years)					33.2 (12.73)	26.33 (5.61)	29.5 (9.18)	ns
Gender (M/F)					4/6	2/7	6/14	ns
Education (years)					12.3 (2.41)	13.44 (2.3)	13.7 (8.46)	ns
Modified Rankin Scale score (score 0–6)					1.1 (0.74)	0 (0)	-	-
[range]					[0–3]	[0]		
Specific treatments of the disease (number of patients)								
	D-penicillamine / Trientine / Zinc				5 / 2 / 0	4 / 1 / 1	-	-
	Liver transplant (hepatic/neurological signs)				3 (2/1)	3 (3/0)	-	-
Other treatments								
	Anxiolytics / Antidepressants / Antipsychotics				3 / 1 / 1	0 / 0 / 0	-	-
Slip lamp examination					8/10	9/9	-	-
(number of Kayser-Fleischer ring at time of diagnosis)					6 with KF ring	2 with KF ring		
Brain MRI (number of patients)					10/10	8/9	-	-
					10 with signs	0 with signs		
Neuropsychological profile								
	Estimated IQ—FNART				109.3 (7.41)	109.22 (5.36)	-	ns
	Total recall score—Word list WMS III				33.67 (6.48)	38.14 (4.78)	-	ns
	Position discrimination—VOSP				28.6 (8.13)	26.22 (9.34)	-	ns
	Number location—VOSP				10 (0)	10 (0)	-	ns
	GZ score—D2				417.2 (50.59)	477.44 (98.77)	-	ns
	KL score—D2				166 (19.66)	185.78 (47.81)	-	ns
	Response time A-B—TMT				42.2 (30.12)	23.78 (7.98)	-	*p* = .07

FNART: French-National Adult Reading Test, WMS: Wechsler Memory Scale, VOSP: Visual Object and Space Perception Battery, TMT: Trail Making Test

### Neuropsychological profiles of WD patients

In order to characterize in a simplified and thus more rapid way the neuropsychological profiles of WD patients, indicators of cognitive functioning were obtained simultaneously with the study.

The FNART (French-National Adult Reading Test [20]) is a list of 36 irregular words following the common rules of pronunciation. It tests the patients’ vocabulary rather than their ability to apply regular pronunciation rules. Scores are transformed to predict premorbid IQ scores. Memory functioning was measured with the “word list” test from the Wechsler Memory Scale-Third Edition (WMS-III, French version [21]). This test includes four learning trials of twelve unrelated words, an interference learning trial of twelve new words, and a delayed recall trial of the initial twelve words 25–35 minutes later. We selected the total recall score–the number of words recalled from the four learning trials–to assess verbal episodic memory.

Visuospatial and visuoperceptual skills were assessed using two tests from the Visual Object and Space Perception battery (VOSP [22]). First, “position discrimination” was assessed with a test that includes twenty cards representing two identical squares with a black dot on them; the subject has to identify the square in which the black dot is exactly in the center. Secondly, “number location” was determined with a test including ten cards with two squares one above the other; numbers from one to nine are randomly distributed within the top square, whereas the other only has a black dot. The participant has to identify which number exactly matches the position of the black dot.

Attention was measured with the D2 test [23], which consists of 14 lines with combinations of d’s and p’s with one to four dashes placed above and/or below the letter; the objective is to mark all d’s with two dashes within 20 seconds for each line. We selected two scores, namely the GZ score–quantitative performance index: total number of marked signs–and the KL score–concentration performance index: total number of correctly marked signs minus incorrectly marked items.

Executive functions and especially cognitive flexibility were measured using the Trail Making Test (TMT [24]). We used the A-B score–calculated as the time difference between TMT-A and TMT-B–since it measures cognitive flexibility independently from manual dexterity.

Non-Neurological and Neurological WD patients did not show significantly different results in neurocognitive functioning (see Table 1). We only observed a trend on the A-B score of the TMT: response time of patients with Neurological WD is slightly longer than the response time of Non-Neurological patients, which is consistent with literature.

Healthy controls did not participate in the neuropsychological examination. Scores obtained by WD patients therefore cannot been compared to this control group. However, compared with norms, scores of WD patients on tests assessing attention and flexibility are, as expected, relatively weak.

### Social cognitive assessments

All participants were tested in a silent room and placed approximately 23 inches from a 15-inch computer screen. Three components of social cognition were assessed with the following tests:

1. The TREF (Facial Emotion Recognition Test, [25]) assesses the ability to recognize six basic and universal emotions (joy, anger, sadness, fear, disgust and contempt). The test includes 54 photos. Facial expressions are represented with color photographs of six different models (three men and three women of different ages). Each photo is displayed during 10 seconds but response time is not limited. Every emotion is presented with nine levels of intensity from 20 to 100%. This assessment provides the overall percentage of emotion recognition (global score), for each emotion (score per emotion) and the level of intensity necessary for each emotion to be recognized with certainty. In this study we specifically analyzed the global score and the scores per emotion.
2. The MASC test (Movie for the Assessment of Social Cognition [26], French version [27]) is a video-based task measuring Theory of Mind abilities. It is a 15-minute movie featuring four people meeting on a Saturday evening. It is meant to analyze affective and cognitive ToM components, and the impairment profiles of the participants, from mentalizing deficit to overinterpretative skills. The movie includes 45 sequences. At the end of each sequence, the subject has to answer a question referring to the actors’ mental states–emotions or feelings, thoughts and intentions–by choosing between four possible responses: the right “ToM” response, the “less ToM” response (undermentalizing), the literal “no ToM” response (no mentalizing at all), or the overinterpretative “excessive ToM” response.
3. The AIHQ (Ambiguous Intentions Hostility Questionnaire [28]; French version by Angelard *et al*., in preparation) measures the hostile social-cognitive bias. The original version of the AIHQ consists of 15 negative situations that differ in terms of intentionality–accidental, intentional or ambiguous situations. In this study, we only used the five ambiguous situations. The subjects were asked to read each situation and imagine the scenario happening to them. Three scores were measured: the “hostility”, “attribution of responsibility” and “aggression” scores. The hostility score was rated by the assessor for each ambiguous situation–from 1, “not hostile at all,” to 5, “very hostile”–according to the participant’s proposition to the question “what do you think was the real reason why the person acted that way?”. The attribution of responsibility score is the average of the participant’s rates on the following three Likert scales: (1) whether the person acted on purpose–from 1, “absolutely not”, to 6, “absolutely on purpose”; (2) how angry it would make the subject feel–from 1, “not angry at all”, to 5, “very angry”; and (3) how much they would blame the other person–from 1, “not at all”, to 5, “very much”. Finally, the aggression score is rated by the assessor–from 1, “not aggressive at all”, to 5, “very aggressive”, for each ambiguous situation–according to the participant’s proposition to the question “What would you do about it?”

### Statistical analysis

Statistical analysis was performed with the Statistica and XLSTAT softwares. Demographic analysis was performed using the Fischer exact test (age and gender) and the Mann-Whitney test (level of education). Given the relatively small sample size, the comparison of the three groups on social cognitive assessments was carried out with the Kruskal-Wallis test, and post-hoc comparison with the Mann-Whitney test.

## Results

### Social cognition

**Fig 1:**
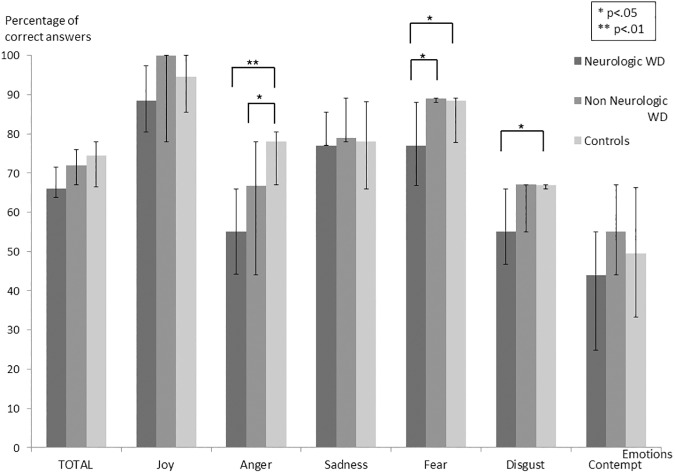
Scores on the TREF assessing emotion recognition.

**Fig 2:**
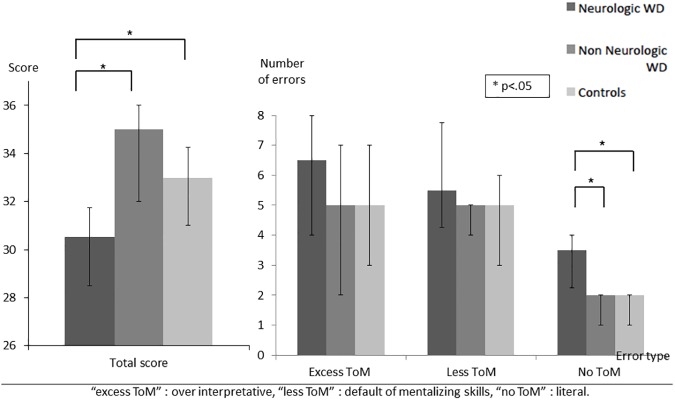
Total score and error type on the MASC test assessing ToM.

**Fig 3:**
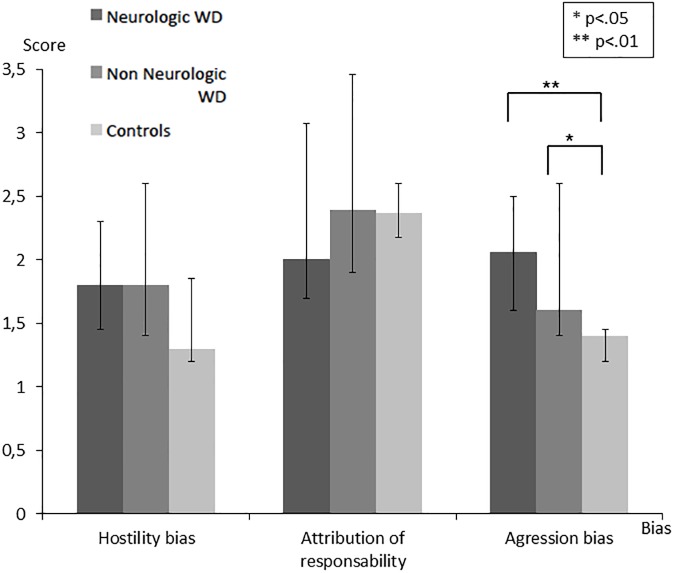
Results on the AIHQ assessing three social cognitive biases.

1. Emotion recognition was assessed with the TREF and showed significant group effects on “fear” (Non-Neurological: med = 89; IQR 88–89 vs. Neurological: med = 77; IQR 66.8–88 vs. Control: med = 88.4; IQR 77.8–89, H = 6.89; *p* = .032), “anger” (Non-Neurological: med = 66.7; IQR 44–78 vs. Neurological: med = 55; IQR 44.1–66 vs. Control: med = 78; IQR 67–80.5, H = 11.89; *p* = .003), and “disgust” (Non-Neurological: med = 67; IQR 55–67 vs. Neurological: med = 55; IQR 46.8–66 vs. Control: med = 66.8; IQR 66–67, H = 5.97; *p* = .05). Post-hoc analyses showed that both Neurological (U = 28.5; *p* = .002) and Non-Neurological WD patients (U = 44.5; *p* = .03) showed significantly lower performance than healthy controls in “anger” recognition. The Neurological WD group performed worse than both healthy controls (U = 50.5; *p* = .03) and Non-Neurological patients (U = 74; *p* = .018) on “fear”. They also had low scores in recognizing “disgust” compared to controls (U = 46; *p* = .017). No significant difference between the groups was observed for “joy”, “sadness”, and “contempt” whereas a trend was observed for TREF global score between Neurological WD (med = 66; IQR 63.8–71.5) and both Non Neurological WD (med = 72; IQR 67–76, U = 23; *p* = .078) and controls (med = 74.5; IQR 66.5–78, U = 141; *p* = .074) (Fig 1).
2. A significant group effect was observed for Theory of Mind (Non-Neurological: med = 35; IQR 32–36 vs. Neurological: med = 30.5; IQR 28.3–31.8 vs. Control: med = 33; IQR 31–34.2, H = 7.90; *p* = .019) on the MASC test. Post-hoc analysis revealed that Neurological WD patients had lower total score than both Non-Neurological WD patients (U = 74; *p* = .019) and healthy controls (U = 45.5; *p* = .018). Regarding error types, a significant effect was observed for errors called “no ToM” (without Theory of Mind), highlighting an impairment in attributing mental states to characters (Non-Neurological: med = 2; IQR 1–2 vs. Neurological: med = 3.5; IQR 2.2–4 vs. Control: med = 2; IQR 1–2, H = 6.73; *p* = .035). Accordingly, Neurological WD patients had a tendency to give more purely literal responses than both Non-Neurological WD (U = 18.5; *p* = .029) and controls (U = 49; *p* = .023) (Fig 2).
3. In attribution bias assessed with the AIHQ, a significant effect was observed for the “aggression score” (Non-Neurological: med = 1.6; IQR 1.4–2.6 vs. Neurological: med = 2.1; IQR 1.6–2.5 vs. Control: med = 1.4; IQR 1.2–1.5, H = 10.08; p = .006). Indeed, Neurological (U = 33.5; *p* = .003) and Non-Neurological WD patients (U = 47; *p* = .041) showed a higher “aggression score” than controls (Fig 3). In contrast, WD patients–both Neurological and Non-Neurological–and controls did not differ in “hostility” or “attribution of responsibility” scores.

### Additional analysis

In the Neurological WD group, a potential confounding effect of anxiolytic, antidepressive and antipsychotic drugs on social cognition was examined by additional analysis. We compared social cognitive scores of the 3 patients who were taking drugs to the 7 patients who did not. We did not observe any significant differences. Indeed, the scores of the 3 patients who were taking drugs were similar to those of the 7 patients who did not (TREF: U = 6.5; *p* = .36, MASC: U = 3.5; *p* = .11, hostility: U = 8; *p* = .57, attribution of responsibility: U = 8; *p* = .57, aggression: U = 10.5; *p* = 1) (S1 File). Moreover, according to some studies, liver transplant in WD yields positive effects on neurological and neuropsychological manifestations [29–31]. In the present study, three patients in each group of WD patients (Neurological vs Non-Neurological) have been transplanted several years before inclusion. These elements limit confounding effects but, to control the impact of transplants, we performed new analyses excluding patients who had received a liver transplant prior to inclusion in the study. We obtained similar social cognitive results compared to those previously highlighted. Indeed, no difference have been demonstrated between Neurological and Non-Neurological WD on both TREF Total score (U = 13; *p* = .25), hostility bias (U = 20.5; *p* = .94), attribution of responsibility (U = 14; *p* = .32), and aggression score (U = 11.5; *p* = .17), but we observed a significant group effect on the MASC Total score (U = 4.5; *p* = .02), as previously observed (S1 File).

## Discussion

To our knowledge, this study is the first to assess several components of social cognition in patients with WD depending on their clinical status–Neurological versus Non-Neurological. We will first present our major results below and then discuss the findings in relation to the three main components of social cognition.

We first observed that patients with WD presented with significant emotion recognition impairments compared to healthy controls. Patients with Neurological WD showed significant deficits in recognizing “fear”, “anger” and “disgust” compared to controls, while patients with Non-Neurological WD only showed impaired “anger” recognition. In the international literature, only one study has reported emotion recognition impairment in WD [17]. This study revealed that patients with WD performed worse than normal controls in identifying “fear”, “anger” and “disgust” from a morphed image continuum based on the photographs of six facial expressions. Wang *et al*. did not however compare WD patient performances in emotion recognition depending on the form of the disease. Our data replicate and specify those obtained by Wang’s team: “anger” recognition seems to be generally impaired in patients with WD, but “fear” and “disgust” recognition appear specifically impaired when WD patients presented with neurological signs.

We observed significant ToM impairment in attributing mental states to characters depicted in a movie in WD patients with neurological disability. Given that the MASC offers the possibility to dissociate overmentalizing from undermentalizing errors, the analysis of error types provides more information than a mere general impairment description. Reduced ToM performances in Neurological WD patients rely on increased undermentalizing errors. This reflects reduced ToM or, in some cases, absence of ToM related to the inability to correctly identify the characters’ attitudes and behaviors. This pattern of results has been extensively described in people with schizophrenia, in which a positive correlation between undermentalizing and negative symptoms has been observed [32].

Finally, regarding attributional style, we observed a significant “aggression bias” in both groups of WD patients–Neurological and Non-Neurological–compared to healthy controls, which means that patients with WD tend to react more “aggressively” to ambiguous social situations than healthy controls (*e*.*g*. they tend to say that they will stop any relationship with a friend if said friend has not called them back one week after they left a message on their answering machine). Clinical observations and experimental studies have shown that WD patients often present with behavioral or emotional disorders, ranging from personality changes, impulsive or instinctive behaviors, to psychotic disorders [16]. Such symptoms have been typically reported in Neurological WD. These patients were considered to have neuropsychiatric disturbances [33]. Changes in the behavior of Neurological WD patients–with increased impulsiveness and aggressiveness–seem to be related to damages to the basal ganglia, which is part of the frontal-subcortical circuits [34]. In our study, Neurological and Non-Neurological WD patients showed a significantly higher “aggression score” than controls. This result is particularly important because it has several possible explanations. First, it is possible that suffering from a chronic disease requiring long-term daily treatment may be associated with attribution bias. In this case, bias in attributional style may be used as a coping strategy, like it has been observed in the field of psychosis [35]. Secondly, it is likely that aggression bias may be an early marker of the occurrence of neuropsychiatric disturbances. Indeed, the aggression score of the AIHQ scale measures the participant’s response to the question: “what would you do about it?” The aggression bias corresponds therefore to a tendency to react aggressively to ambiguous situations. In WD, psychiatric features include emotional lability, impulsiveness, disinhibition, and self-harming behavior [16]. These psychiatric symptoms had been typically thought to be present along with neurological symptoms–and patients were labeled as having neuropsychiatric disturbances [33]. The present study reports an aggression bias in a group of WD patients without neurological signs of the disease. We assume that aggression bias could be linked to very fine frontal signs such as irritability or impulsiveness, and be an early marker of neurological damages. This trend from Non-Neurological to Neurological WD patients may represent an additional indicator of a sub-clinical finding in Non-Neurological patients, that may progress and motivate longitudinal research. Actually, it would be interesting to assess this component in Non-Neurological WD patients during two or five years, and measure the occurrence of neurological signs according to the evolution of this bias and compliance with the treatment. Finally, one may hypothesize a link between this bias and emotional impairments. Actually, difficulties of WD patients in emotion recognition, in particular with regard to negative emotions, could have an impact on the way they perceive ambiguous situations in daily life.

While cognitive deficits have been widely documented in patients with Neurological WD, our study emphasizes the presence of social cognitive impairments in the disease for the first time. Brain areas involved in social cognition are referred to as “the social brain”, which includes the amygdala, orbital-frontal cortex and temporal areas [11] but also or more precisely the medial prefrontal cortex, the superior temporal gyrus and the temporoparietal junction [12;36–37]. Brain damage in WD primarily involves the basal ganglia [38] and particularly, in decreasing order of prevalence, the putamen, the globus pallidus, and the head of the caudate nucleus; they seem to be central to explain the cognitive deficits observed in WD [3]. Recent brain MRI studies have found that other parts of the brain may also be affected, such as cerebellar peduncles, the corpus callosum and corticosubcortical white matter with frontal predilection [39–41]. Moreover, the basal ganglia are indeed a highly-organized network with a major cortical projection, which explains why they are involved in movement control as well as in associative learning, executive functions, working memory and emotions [42–44]. According to Cummings [34], personality and behavior disorders–which correspond to increased impulsiveness and aggressiveness–and affective disorders may result from damages to the basal ganglia because of their involvement in the frontal-subcortical circuit. In WD, this hypothesis was also studied by Seniów *et al*. [16]. WD patients with basal ganglia lesions exclusively showed more marked inadequate control of affective behavior than both healthy controls and WD patients with multifocal damages. In our study, no specific analysis could be performed regarding correlations between social cognitive scores and brain MRI abnormalities due to the small number of subjects. Now that social cognitive impairments in WD have been explored, it would be interesting to conduct an exhaustive investigation of social cognition in relation to lesions involving the basal ganglia.

To conclude, this study shows for the first time that WD patients with neurological signs and, to a lesser extent, hepatic signs present with impairments in several domains of social cognition. The impact of our data has nevertheless some limitations due to both small sample size and heterogeneity of our WD population in terms of evaluations and therapies. Moreover, we did not control WD patients for psychiatric comorbidities (except for depression). Some factors such as anxiety or even the fact that suffering from a chronic disease could have a negative impact on social cognitive functioning. Moreover, impact of psychotropic medication on social cognition could be mentioned as limitation, as well as liver transplant, even if these factors did not seem to affect our data. However, the sample size of this study did not allow a definitive conclusion regarding these questions. Finally, contrary to several studies, we did not observe significant differences of neurocognitive functioning between Neurological WD patients and Non-Neurological WD patients in this work. This contradictory finding seems to be due to the fact that we used a reduced neurocognitive battery, unable to assess all components of neurocognition, usually impaired in WD. Our study yet opens further research to confirm social cognition impairments in WD patients depending on their clinical status, to analyze the relations between social cognition impairments and alterations of other cognitive processes (especially executive functions), and to better understand the links between the cortical and subcortical areas involved in social cognition and the brain lesions observed in WD. Since cognitive remediation programs targeting social cognitive impairments have recently been developed, it would be interesting to assess the potential effectiveness of such therapies on social cognitive impairments in WD and their impact on social behavior.

## Supporting information

S1 File Supporting information. (XLSX)
